# Patient, supporter and primary healthcare professional perspectives on health risks in over 16s with attention deficit hyperactivity disorder (ADHD) in England: a national survey study

**DOI:** 10.1186/s12913-024-11188-5

**Published:** 2024-06-19

**Authors:** John H. Ward, Kieran Becker, Jane Smith, Anna Price, Tamsin Newlove-Delgado

**Affiliations:** 1 University of Exeter Medical School, 2.05 South Cloisters, St Luke’s Campus, Heavitree Road, Exeter, UK; 2 Department of Psychiatry, University of Oxford, Oxford, UK; 3 Royal Devon University Hospital NHS Foundation Trust, Devon, UK; 4 Oxford Health NHS Foundation Trust, Oxford, UK

**Keywords:** Attention deficit hyperactivity disorder, Qualitative research, Primary care, Preventative medicine, Health behaviour, Qualitative research

## Abstract

**Background:**

Current research suggests that people with attention deficit hyperactivity disorder (ADHD) are at higher risk of physical and mental health disorders. This study aimed to explore these health risks in ADHD from the perspectives of multiple stakeholders.

**Methods:**

This study forms part of the ‘Managing young people with ADHD in Primary care (MAP) study’. A survey developed by the study team was distributed to over 16 year olds with ADHD, their supporters, primary healthcare professionals and health commissioners across England, via social media and through patient/clinical networks (September—October 2022). This survey contained two questions on health risks. Question one asked about views on health risks in ADHD (free text). Question two asked about advice given (options list and free text). Descriptive statistics summarised responses to questions one and two, and qualitative analysis (reflexive thematic analysis) was used to explore free text responses from question one.

**Results:**

782 participants responded to the MAP survey. Of these, 206 healthcare professionals, 157 people with ADHD and 88 supporters answered question one. The most mentioned perceived risks were substance misuse, sleep disorders, weight management and smoking. More people with ADHD reported disordered eating as a health risk (*n* = 32) than healthcare professionals (*n* = 5). Generated themes included perceived health risks, impact of living with ADHD, lack of adequate healthcare, and need for ADHD awareness. In respect to advice given (question two), based on responses from 258 professionals, 162 people with ADHD and 100 supporters, the most common advice discussed in consultation was mental health (*n* = 149, *n* = 50 and *n* = 17 respectively). High numbers of respondents reported not giving/receiving advice on wider health (*n* = 38, *n* = 88 and *n* = 61 respectively).

**Conclusions:**

Findings demonstrate that respondents perceived a range of physical and mental health risks posed by ADHD. These related to difficulties with activities of daily living, as well as healthcare interactions and the impact of core features of ADHD (e.g. impulsivity, emotional dysregulation). These risks are not currently explicitly addressed in United Kingdom national guidance on ADHD. More work is needed to examine and address the broader health outcomes of people with ADHD.

**Supplementary Information:**

The online version contains supplementary material available at 10.1186/s12913-024-11188-5.

## Background

Attention deficit hyperactivity disorder (ADHD) is a neurodevelopmental condition characterised by core features of hyperactivity, inattention, and impulsivity. Often diagnosed in childhood, approximately 65% of children diagnosed with ADHD will have persistence of symptoms into adulthood [1]. People with ADHD are thought to experience a range of poorer socio-economic, physical, and mental health outcomes in later life, with an estimated average 15-year reduction in life expectancy from predictive modelling [2–4].

There is a growing literature surrounding the broader health of people with ADHD. Research demonstrates that people with ADHD are at greater risk of multiple potential health problems covering different systems and aetiologies including medication side effects (e.g. hypertension), alcohol/substance abuse, types 1 and 2 diabetes, neurological disorders such as epilepsy and migraine, respiratory conditions (e.g. chronic obstructive pulmonary disease, asthma), sleep problems and weight management difficulties [5–19]. Beyond these physical health risks, the mental health impact of ADHD is well-established, with higher rates of affective disorders and personality disorder than seen in the general population [20, 21].

These poorer health outcomes are likely to be influenced by an interplay of genetic, environmental, behavioural, psychological, structural, and service-related factors [22–28]. As suggested by DuRietz et al., genetic and sibling studies show that behavioural explanations are insufficient to explain the increased rates of non-communicable disorders in those with ADHD [11]. Behavioural factors are likely to make a significant contribution to certain problems, for example, studies suggest people with ADHD are more likely to have dental caries [13, 14]. The significant association of ADHD with executive function difficulties could theoretically make activities of daily living (ADLs) more challenging, which in turn may affect physical health indirectly (e.g. coping mechanisms, self-care activities) and directly (e.g. difficulties with healthcare engagement, chronic disease management) [26, 29–31]. There are also other important factors at play, for example the impact of chronic stress related to ADHD on long-term physical and mental health, both directly (e.g. neuroendocrine hypotheses) and indirectly (e.g. financial impacts of sickness) [28, 32–34].

Socioeconomic factors—a correlate of both broader health and ADHD—have also been implicated in the relationship between ADHD and physical health. Landes and London, in their study of the health of nearly 20,000 adults in the United States (US) with ADHD, found that socioeconomic factors, whilst not completely explaining the relationship between physical health and ADHD, were significantly implicated [22]. For young people and adults with ADHD, there are also further challenges. Firstly, people with ADHD are more likely to be lost in the transition between child and adult mental health services [25]. Without this continuity in care, messages around health and possible support within an ADHD context may not be provided. Secondly, adulthood presents the challenge of greater independence/less support (e.g. moving out of the family home, going to university, employment) and increased psychological demands (e.g. financial responsibilities, more demanding workload). This may lead to both maladaptive coping strategies and more conceptual priorities such as long-term health being neglected [27, 28, 35]. People with ADHD therefore experience poorer physical and mental health outcomes, compounding existing inequalities and leading to associated social and economic costs for the individual, family, and society. Addressing this spectrum of increased risks is likely to require a broad approach, ranging from prevention and health promotion to more specific or tailored interventions amongst people with ADHD.

There are some health interventions that have demonstrated promise in relation to the wider health of people with ADHD. For example, psychotherapy and psychoeducation in relation to physical health problems has been tried in groups of adults to improve knowledge about health and self-efficacy [36–41]. There have also been studies in the context of alcohol and substance misuse, showing response to stimulant medication when substance use disorder co-occurs with ADHD [42–44]. However, the evidence base is small and heterogenous, and therefore these findings have likely not permeated into clinical practice. This is reflected in UK National Institute for Clinical and Health Care Excellence (NICE) guidance on ADHD— which alludes to having broader discussions with patients about healthy lifestyles and alcohol/drugs, but which does not include information on specific risks or recommendations for tailored advice [33].

To design and implement prevention or intervention approaches to address general health risks associated with ADHD, it is important to understand how key stakeholders perceive these risks, in terms of their awareness, conceptualisation and experiences. These perspectives are likely to influence the effectiveness of any policies, services or interventions aimed at health professionals and people with ADHD. However, to the authors’ knowledge there is no literature which specifically explores awareness of health risks in people with ADHD. Whilst a few studies have explored primary care professionals’ perceptions of ADHD in general, again none have focussed on awareness or perceptions of broader health risks [45, 46].

Primary care professionals have a particularly important part to play in identifying and addressing health risks, as they provide holistic and accessible first line care for common and long-term conditions [46]. Additionally, in England, they increasingly prescribe and monitor ADHD medication under shared care arrangements, which involve aspects of physical health monitoring [25, 46, 47]. This study therefore aimed to explore awareness and perceptions of health risks in ADHD from the perspectives of people with ADHD, supporters and primary healthcare professionals in England.

## Methods

This study forms part of the National Institute for Health Research (NIHR)-funded Mapping ADHD services in Primary Care (MAP) Study [48–50]. The MAP study has three phases (surveys, qualitative interviews, and co-production). This paper presents analyses of a sub-set of data from the phase one mapping survey, an online survey of people aged over 16 years old with ADHD, supporters of people with ADHD, primary healthcare professionals and health commissioners. The analyses focussed on responses to two health risk questions in the survey (see Supplementary Material S1) and included quantitative (aimed at summarising identified health risks) and qualitative approaches (to explore perspectives on risks). These health risk questions were not posed to health commissioners (*n* = 42), and therefore whilst they received the survey they are excluded from our analyses (see related publication [49]).

### Participants

Survey participants providing data for these analyses fell into three categories: people (aged over 16s) with ADHD, supporters, and primary care professionals.

Inclusion criteria for the lived experience group were; aged over 16, lived experience of ADHD (with or without diagnosis) and residing in England. Supporters had to be supporting someone with ADHD aged over 16 years old. Primary care professionals had to have a paid or voluntary role in primary care (clinical or non-clinical), and work or live in England.

### Recruitment

The recruitment strategy is described in detail in the protocol paper for this study, and was based upon both purposive and convenience sampling [48]. This is a purposive sample, in that geographic variation in the advertising of the survey was deliberately introduced, but follows convenience sampling in who went on to access the survey, given the efficiency of convenience sampling in gathering a large sample size in the timespan of this project. This sample was reached through advertising via social media (X (formerly known as Twitter), Instagram and Facebook), national ADHD groups/platforms (e.g. ADHD Foundation, UK Adult ADHD Network), and promotion via local primary care networks, local health education administrator distribution lists (i.e. general practice (GP) training administrators) and regional academic networks (e.g. South West Clinical Research Network).

The survey was open for approximately six weeks, between October and November 2022. The minimum response target was 252 participants. This was based on a minimum of 6 responses for each of the 42 Integrated Care Systems (ICSs) in England [48].

To aid with full completion of surveys, participants were able to enter a prize draw for a £50 voucher on completion of the survey. This was stated at the start of the survey but not advertised alongside the survey, so as to reduce the risk of non-genuine participants.

### Survey design

Data were collected via online surveys. These were created by the study team, building on previous work and following the seven-step method for stakeholder informed service mapping [51, 52]. Although not a validated measure, these surveys were developed (i.e. conceptualisation and creation of questions) and piloted with input from professionals and over 16s through the study’s research advisory groups, and were suitable to address our research questions. The surveys were tailored for each group (e.g. HP and LE) and included questions on a range of topics relating to management of ADHD in general and within primary care specifically, including availability of support, prescribing, and awareness of health risks. The surveys were trialled with different stakeholder groups to ensure accessibility and face validity. The HP and LE surveys were constructed in Qualtrics [53].

This study explores responses to two questions in the HP and LE surveys, about health risks to participants (see Supplementary Material S1). The first question asked, “*What do you consider to be the most important increased health risks linked with having ADHD?”.* This question was entirely free text response. The second asked participants to select what health advice they have given or received, specifically asking about; sexual health, smoking, physical activity and healthy eating, long-term physical/mental health problems, risky behaviour (including substance misuse), but also included a free text response option (other). This combination of questions was asked in order to encourage open responses from participants, but with the second question being more directive in understanding what advice may be given in healthcare consultations. Participants were also free to enter their own responses here. The exact questions can be found in supplementary materials (Supplementary Material S1), alongside the full survey (Supplementary Material S1).

### Data analysis

Data were extracted from Qualtrics, collated and cleaned. Participants who did not meet the inclusion criteria, or who left the survey before identifying their role (i.e. person with ADHD, supporter, primary care professional), were excluded from the final dataset. For these analyses, incomplete survey responses were not excluded, as long as respondents had answered the questions of interest.

The analyses used quantitative and qualitative approaches, as the questions elicited both shorter responses listing health problems or issues, and longer and richer free text responses.

### Free text coding/Quantitative analysis

Responses were tabulated in Microsoft Excel, repeatedly read and coded for mentions of different health risks (e.g. weight management, smoking, addiction), using a binary response option (i.e. either 1 (mentioned) or 0 (not mentioned)). Mentions of one health issue were only coded once for each respondent. Coding used an iterative deductive approach, where initially a broad approach was taken to coding which was then reduced into smaller and more meaningful categories (e.g. mentions of different risks around drugs and addiction were reduced into a broader category of substance misuse). These categories were discussed and refined within the study team (TND, JW, AP). Codes were summarised quantitatively, and frequency reported by stakeholder group (e.g. person with ADHD/supporter or primary care professional).

For the second multiple choice question regarding advice given/received, free text responses were manually coded and incorporated into the frequency data from the multiple choice.

### Qualitative analysis

All free text responses were screened to identify those which did not simply list health risks and included further free text data. These data were used in the qualitative analysis. NVivo 1.7.1 was used to manage the qualitative data [54]. Given the novelty of the field, an inductive approach was employed, using the approach to reflexive thematic analysis described by Braun and Clarke and adopting a critical realist framework [55–57]. After familiarisation with the dataset, in part assisted through the processing of quantitative analysis as described above, data were initially coded line-by-line by JW (a medical physician) to create a coding frame. The frame was then iteratively reviewed and refined through application to the data and discussion with the team (with mixed backgrounds, including both professional and lived ADHD experience) (JW, AP, TND, KB). Themes were then generated from the data, developed and reviewed, considering coherence and consistency. JW kept a regular log of analytical notes and questions throughout the analysis and reflecting on differences/connections between data, and on the impact of researchers on the analysis e.g. for those with clinical backgrounds, there was a need to maintain an open approach rather than minimising or dismissing comments by participants which might contradict our ‘professional’ understanding.

## Results

### Quantitative analysis

In total, there were 782 responses to the MAP survey, including 42 responses from commissioners who did not answer questions about health risks and are not included in the following data (see related publication for details [49]). There were 740 responses from people with ADHD, supporters and healthcare professionals. Of these, 331 (44.7%) were primary healthcare professionals, 238 (32.2%) were people with ADHD, and 171 (23.1%) were supporters for people with ADHD. The largest group of respondents were general practitioners (*n* = 197), followed by people with ADHD over the age of 26 (*n* = 177). In total, 522 respondents provided an answer to question one (health risks) and/or question two (health advice). For details of response numbers to the MAP survey and questions of interest, by stakeholder group, see Table 1.

**Table 1:** Table detailing stakeholder groups responding to the MAP study survey, and questions of interest for these analyses

Respondent type (broken down)	Number of respondents (across MAP surveys)	Respondents to question 1 (Health Risks) (n = 451)	Respondents to question 2 (Health Advice) (n = 520)	Responses to question 1 and/or 2 (n = 522)
**Primary Care Professionals**	**n = 331**	**n = 206**	**n = 258**	**n = 258**
General Practitioner	197 (59.5%)	160 (77.7%)	185 (71.7%)	185 (71.7%)
Nurse	48 (14.5%)	21 (10.2%)	32 (12.4%)	32 (12.4%)
Manager or administrator	32 (9.7%)	8 (3.9%)	18 (7.0%)	18 (7.0%)
Other role in primary care	46 (13.9%)	17 (8.3%)	23 (8.9%)	23 (8.9%)
Role not given	8 (2.4%)	0	0	0
**People with ADHD**	**n = 238**	**n = 157**	**n = 162**	**n = 163**
ADHD Age 16–25	57 (23.9%)	35 (22.2%)	36 (22.2%)	37 (22.7%)
ADHD Age 26+	177 (74.4%)	122 (77.7%)	126 (77.8%)	126 (77.3%)
Did not answer	4 (1.7%)	0	0	0
**ADHD Supporters**	**n = 171**	**n = 88**	**n = 100**	**n = 101**
Supporter of person with ADHD Age 16–25	116 (67.8%)	73 (83.0%)	83 (83.8%)	84 (83.2%)
Supporter of person with ADHD Age 26+	33 (19.3%)	15 (17.0%)	16 (16.2%)	17 (16.8%)
Role not given	22 (12.9%)	0	0	0
Commissioners*	**N = 42**	NA	NA	NA
**Total**	**782**	**451**	**520**	**522**

*Note: commissioners were not asked questions 1 or 2, so commissioner data is not included in our analyses

### Health risk identification

In relation to the question on health risk identification, responses were received from 206/331 (62.2%) healthcare professionals, 157/238 (66.0%) people with ADHD and 88/171 (51.5%) supporters. The statistics provided below are based upon the entire sample eligible to answer the question (i.e. 740 participants, from 331 professionals, 238 people with ADHD and 171 supporters).

**Fig. 1:**
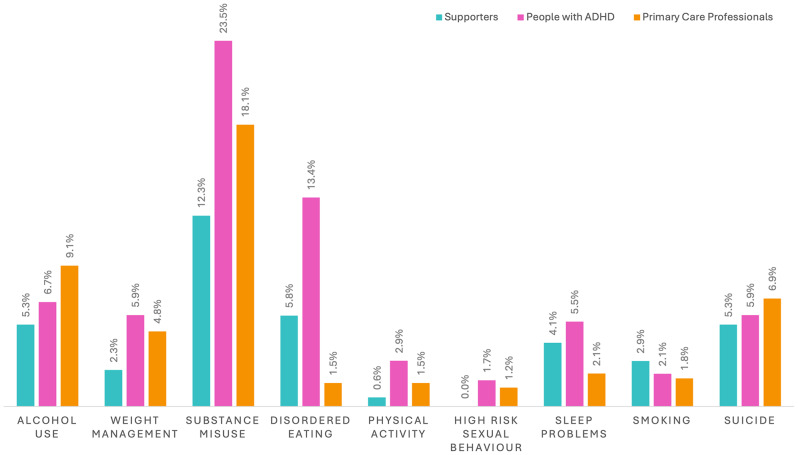
Bar chart demonstrating the frequency of mentions of each health risk, as a percentage of survey respondents from each stakeholder group (331 healthcare professionals, 238 people with ADHD, 171 supporters)

The frequencies of the physical health risks mentioned are shown in Fig. 1. When responses were combined across stakeholder groups, substance misuse was the most mentioned topic, with 137/740 (18.5%) perceiving drug use as a physical health problem amongst people with ADHD. The other frequently mentioned risks were alcohol (55/740, 7.4%) and disordered eating (47/740, 6.4%).

Disordered eating was the second most mentioned problem amongst people with ADHD, whilst being amongst the least frequently mentioned topics for healthcare professionals and supporters of people with ADHD. The least frequently mentioned risks overall included smoking (16/740, 2.16%), physical activity (13/740, 1.76%) and high-risk sexual activity (8/740, 1.08%).

### Healthcare advice

The results are presented in Table 2. In respect to advice given/received, responses were received from 259/331 (78.2%) healthcare professionals, 162/238 (68.1%) people with ADHD and 100/171 (58.5%) supporters. Overall, healthcare professionals reported giving advice far more than those with lived experience of (people with ADHD and their supporters) reported receiving it. The most common topics which professionals reported advising on were mental health (*n* = 149/331, 45.0%) and smoking (*n* = 136/331, 41.1%). Just under a quarter of GPs reported advising on sexual health (*n* = 79/331, 23.4%).

**Table 2:** Table detailing health advice given. Percentages based upon denominators given in group column

Group	Advice on mental health	Advice on risky behaviours (including substance misuse)	Advice on chronic physical health conditions	Advice on physical activity and healthy eating	Advice on quitting smoking	Advice on sexual health (including screening)	Don’t Know	No advice provided
**People with ADHD (n=238)**	50 (21.0%)	8 (3.4%)	14 (5.9%)	19 (8.0%)	10 (4.2%)	14 (5.9%)	1 (0.4%)	88 (37.0%)
**Supporters (n=171)**	17 (9.9%)	7 (4.1%)	17 (9.9%)	12 (7.0%)	10 (5.9%)	8 (4.7%)	7 (4.1%)	61 (35.7%)
**Healthcare Professionals (n=331)**	149 (45.0%)	107 (32.3%)	128 (38.7%)	127 (38.4%)	136 (41.1%)	79 (23.9%)	42 (12.7%)	38 (11.4%)

By contrast, mental health and the management of long-term physical health problems were the areas most advised on from the supporter perspective. From the patient perspective, mental health was mentioned by 21% of respondents (*n* = 50/238), with other items being reported by under 10%.

Furthermore, a large number of people with ADHD and their supporters reported not being provided with wider health advice (88/238 (37%) and 61/171 (35.7%) respectively). Conversely, 38 (11.5%) primary care professionals reported that wider health advice was not provided by their service.

There were fewer free text responses to this question, therefore these are summarised below, and these data were not analysed qualitatively. For people with lived experience of ADHD, responses relayed difficulties in accessing services signposted to due to ADHD, misdiagnosed ADHD and help with the management of chronic health conditions. For healthcare professionals, responses alluded to exploring preventative care with all patients irrespective of ADHD, greater social advice (e.g. housing, finances) and point out that there are no ADHD-targeted health services.

### Qualitative analysis

**Fig. 2:**
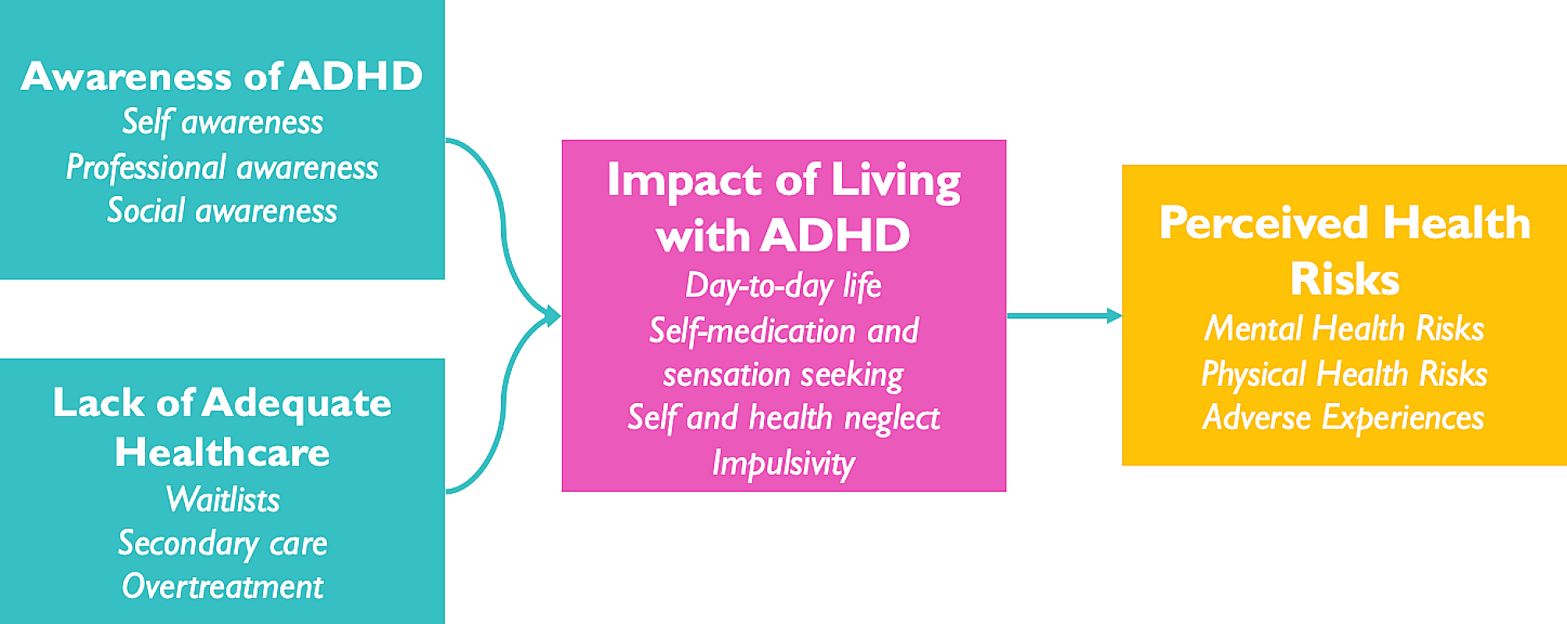
Graphical representation of the qualitative themes generated in the thematic analysis

There were sufficiently rich extracts to analyse free text responses from 36 people with ADHD, 26 Healthcare professionals, and 21 supporters. This data therefore represents the perspectives of a smaller and self-selected (e.g. they filled in the open text response boxes) subset of the 740 HP and LE respondents to the MAP survey. The themes generated from free text responses are displayed in Fig. 2. These were: *Perceived Health Risks, Impact of living with ADHD, Lack of Adequate Healthcare* and *Awareness of ADHD.*

### Perceived health risks

This theme relates to the health problems explicitly identified by people with ADHD, supporters, and professionals. These fell into physical health risks, mental health risks and adverse experiences which have the propensity for harms to health (e.g. homelessness).

In respect to physical health, participants mentioned cardiovascular risks from prescribed medication, but also referenced high blood pressure and tachycardia related to anxiety and the impact of this on their health. One participant perceived that a lack of support and understanding had also had consequences for their health:

*“I’ve recently had cancer which I believe came from binge eating and inflammation of my body from being misunderstood and supported with ADHD in females”* (*person with ADHD*)

In relation to mental health, ‘burn out’ was referenced frequently by people with ADHD, as were low self-esteem and stress.

*“It is just survival and it is hard and scary”* (*person with ADHD*).

Anxiety and depression were also perceived as common risks to health, and often referenced in relation to poorer physical health.

Participants also perceived adverse experiences to be a risk of ADHD. One person with ADHD referenced ‘propensity for traumatic situations’ which encapsulates this theme well—the notion raised by participants that having ADHD carries intrinsic social risk. Participants (particularly people with ADHD and healthcare professionals) described perceived risks including criminal activity, prison, abusive relationships, financial difficulties and homelessness:

*“I work at a specialised homelessness practice. We have a higher than average proportion of people with substance use disorders and homelessness with a diagnosis of ADHD” (healthcare professional)*.

### Impact of living with ADHD

This theme explores the difficulties people with ADHD may encounter in day-to-day life, and how this may relate to their health. As one person with ADHD put it, “*ADHD makes practically everything about making a living harder*”. The theme was sub-divided into; awareness of ADHD, lack of adequate healthcare, impact of living with ADHD and perceived health risks.

Participants mentioned finding difficulties in basic activities of life such as cooking or hygiene. Some people with ADHD also perceived that they had to exert themselves much harder to do the things that their neurotypical peers could do with ease, thus impacting on their mental health and their ability to manage activities such as exercise or making medical appointments. This links into the subtheme of self and health neglect, where participants found self-care activities difficult.

*“I do not cook as it is too much effort, or I burn stuff, forget it’s cooking or set myself in [sic] fire*” (*person with ADHD*).

People with ADHD reported forgetting or neglecting to make/attend healthcare appointments, which healthcare professionals also perceived as a risk. The concept of time management and prioritisation was repeatedly mentioned by people with ADHD in reference to this, such as leaving things to the last minute, ‘time blindness’ and forgetfulness.

Some participants then went on to reference behaviour which they appeared to view as overtly impacting health. These behaviours seemed to fall into two subthemes. The first theme was ‘impulsive behaviour’, where people with ADHD reported they would ‘act impulsively’ or ‘become impulsive’ and this would lead to a health risk, for example substance misuse, smoking, sexual activity, reckless driving or ‘taking risks’ (or words to that effect). Participants did not reflect a particular emotional regulation function in these statements. One quote that exemplifies this subtheme is:

“*With regards to physical health, my impulsiveness was probably putting me at more risk in general” (person with ADHD)*.

Under a second, self-medication and sensation seeking subtheme, participants would mention behaviours associated with health risks that appeared to serve a specific emotional or regulatory function. For example, substance misuse as a form of self-medication, ‘dopamine seeking’ and ‘stims’ were referenced:

*“…food is a battleground, an enemy, an addiction. I understand now that it’s probably a stim for me, as well as an instant easy dopamine hit for my ADHD brain” (person with ADHD)*.

### Lack of adequate healthcare

This theme denotes difficulties experienced by people with ADHD, professionals and supporters in relation to the care of people with ADHD, and potential consequences in terms of their impact on health. The subthemes generated were; waitlists, secondary care and overtreatment.

As expected, participants frequently referenced long waiting lists for assessments for ADHD on the NHS. People with ADHD mentioned the lack of support available to them in the interim whilst awaiting a diagnosis.

*“…the parlous state of service provision. In our county there is a 2 year plus wait for an NHS appointment. Following assessment, a SCP [shared care protocol] is issued but there is very little ongoing follow up” (healthcare professional)*.

*“I feel like I’ve just been left to manage my symptoms alone” (person with ADHD)*.

They also talked about then going onto seek private diagnoses, or via right to choose legislation. The issues of there not being appropriate services to refer into was also raised.

Healthcare professionals discussed the problems with secondary care, and in particular shared care agreements. As one GP put it, ‘primary care are left holding the baby’. Some described frustration that specialist services place significant burdens of prescription management onto primary care. People with ADHD echoed frustrations about waiting for prescriptions post-diagnosis or having to pay for private prescriptions.

Additionally, the issue of overtreatment was raised by a supporter of a person with ADHD and a GP, alluding to the side effects from medications causing harm and whether this balances with the benefits of diagnosis.

*“There is likely to be a lot of false diagnosis and unnecessary treatment of patients” (supporter of person with ADHD)*.

*“Hardly anyone seen by the private provider comes back without a diagnosis of ADHD” (healthcare professional)*.

### Awareness of ADHD

This theme draws heavily on the experiences of people with ADHD, but also has viewpoints from supporters and healthcare professionals. The subthemes were self-awareness, professional awareness and social awareness.

Under the self-awareness subtheme, people with ADHD reflected several problems; not understanding their own ADHD, not knowing that they had ADHD, or revisiting an early ADHD diagnosis in the context of new adult difficulties. Some participants also discussed belongingness, and how their ADHD can make them feel alienated.

*“it took a lot of therapy before I revisited my ADHD diagnosis to understand how it affects me as an adult” (person with ADHD)*.

In terms of professional awareness, some people with ADHD and their supporters reflected that they felt their primary healthcare professionals did not understand ADHD, how it may affect them or what support they may require, and perceived that this in turn had an impact on their physical and mental health.

*“I’ve found it difficult to get additional help for the combined issues of having ADHD and perimenopause at the same time” (person with ADHD)*.

Some health professionals also reflected that they were underinformed about ADHD or had limited interactions in this realm.

*“I do not have enough knowledge around ADHD, possibly a learning point for the practice” (healthcare professional)*.

Some people, particularly supporters, also expressed frustration at education professionals, whom they felt did not understand the difficulties their children experienced and the impact that had on children.

Some people with ADHD felt that friends, family or employers did not understand their ADHD and how it affected them, and that this wider lack of social understanding affected their physical or mental health.

*“Personally I feel that having ADHD has impacted my mental health because of how other people treat me” (person with ADHD)*.

## Discussion

Four key themes were generated from reflexive thematic analysis; perceived health risks, impact of living with ADHD, lack of adequate healthcare and ADHD awareness. These themes expand on the identified health risks, both mental and physical, to people with ADHD. The findings can help with understanding how professionals and people with lived experience of ADHD view health risks, which can inform the development and implementation of prevention and intervention efforts.

From our survey responses, the most mentioned health risks were drugs and substance misuse. However, some identification of risks did vary by group. For example, disordered eating was the next most mentioned risk for those with lived experience, (mentioned by 13.5% of people with ADHD and 5.9% of supporters) while for healthcare professionals, this was amongst the lowest reported risks (1.5%). Other areas of difference were sleep (mentioned more by people with ADHD and supporters than healthcare professionals), sexual activity (not mentioned by supporters), physical activity (mentioned more by people with ADHD than any other group) and weight management (mentioned more by people with ADHD and healthcare professionals than supporters). While these differences may stimulate speculation on differing priorities of each stakeholder group, given the nature of the survey sample and open nature of questions it is difficult to draw firm conclusions.

Substance use was by far the most identified risk in quantitative analysis, and this was consistent amongst groups. The qualitative findings also echo that participants perceived the risks of use of substances as part of impulsivity and ‘self-medication and sensation-seeking’ in ADHD. This latter theme, where participants talk about behaviour with specific functions (e.g. stimulation, self-medication) possibly relates to emotional dysregulation. Emotional dysregulation has been linked to some of the health risk behaviour that is identified by participants in this study, especially alcohol/substance misuse, smoking and risk-taking in an ADHD context [58–61].

Another prominent theme in the qualitative analysis from all stakeholder groups was “lack of adequate healthcare”, indicating that this was seen as a health risk alongside more ‘concrete’ risks such as substance use. It is interesting to note that whilst people with ADHD and supporters said that they felt unsupported and unmanaged, primary care professionals reflected difficulties in both engaging with people with ADHD and dealing with secondary/private care. Concerns over the provision of healthcare for ADHD have been consistently raised, including continued difficulties in accessing timely diagnosis and treatment [47]. Without clear guidance or good existing infrastructure, it makes sense that generalists will struggle with the holistic management of ADHD. The difficulty in managing patients with physical and mental health concerns is not unique to ADHD; a study of clinicians in a UK-based community mental health team (CMHT) found that many expressed uncertainty around managing the physical health of their patients. A qualitative study of Irish GPs also highlighted challenges in engaging patients with mental health problems in their physical healthcare [62, 63].

Weight management (both being underweight and overweight) also was a commonly identified health risk, mentioned by around 4–6% of people with ADHD and healthcare professionals. In the wider literature, difficulties with weight management in relation to ADHD have been observed both in genetic studies and epidemiological studies [7, 64–67]. This raises the question of whether weight management needs to be highlighted more explicitly to people with ADHD, supporters, and healthcare professionals in respect to ADHD. However, weight management does not currently appear in UK ADHD guidance, possibly due to the lack of evidence-based interventions in this field. While there is research around the impact of medication on BMI, very few studies explore the potential of ADHD specific weight interventions, beyond one small pilot study (Bjork et al., 2020) [38, 64].

Some of the findings of our analyses were unexpected. Disordered eating appeared to be much more commonly identified as a risk by people with lived experience than by primary care professionals. Two of the subthemes within ‘the impact of living with ADHD’ addressed disordered eating; day-to-day life (referencing ADHD affecting ability to eat regular meals, prepare food etc.), as well as self-medication and sensation-seeking (referencing food as a ‘stim’ and binge-eating). These themes reflect reporting of a disordered relationship with food, which is consistent with findings elsewhere in literature [68–73]. However, healthcare professionals in this survey rarely reported disordered eating as a health risk. One possible explanation for this difference may be the significant prevalence of disordered eating in the general population and the poor detection of those in need [74, 75]. However, the unprompted mentions of disordered eating by people with lived experience underline that this is perceived as an important health issue in the population studied, which is currently not referenced in national guidance [76].

Although consistent between participants, smoking and sleep problems were less often identified by respondents than one would expect, given their prevalence in the ADHD literature [5, 9, 42, 77–81]. Interestingly, neither sleep nor smoking are covered in current UK guidance [76].

The frequent references to mental health (particularly anxiety, depression, and low self-esteem) amongst responses, where mention of physical health risks had been anticipated, were also unexpected, but perhaps demonstrate a recognition by respondents of the interplay between mental and physical health risks. Some participants referenced self-esteem as a contributor to poor mental health, but others went further in describing low self-esteem as a reason why they may neglect their health or partake in risky behaviour. ADHD has been previously linked with lower self-esteem, and furthermore implicated by qualitative research as a factor in the lifetime adverse experiences of young people and adults with ADHD (e.g. difficulties in school, peer interactions) [3, 82, 83]. It is also known that self-esteem relates to physical health and self-care behaviour independently of ADHD [84, 85].

Another surprising finding was the prominence of adverse experiences (e.g. homelessness, poverty, violence) in responses to a question on health risks. Whilst ADHD is associated both with adverse childhood experiences as well as long-term poorer social and occupational outcomes [48, 67, 72], it is interesting that both people with ADHD and professionals in our survey clearly recognised the impact of these wider social factors on health.

With a complex and multi-impact condition such as ADHD, it could be argued that focussing on individual health risks has limited utility. Whilst some of the health risks identified may be mainly neurobiological, and less affected by the cultural and social context (e.g. sleep disorders, weight [42, 67, 86]), many health risks (e.g. substance misuse, self-harm, high risk sexual behaviour) are more likely to relate to the underlying neuropsychological processes in ADHD such as difficulties in evaluation of risk and delay discounting [87–89] and associations with impulsivity [90–99].

The translation of these neuropsychological differences to the commonplace is well-demonstrated in the ‘impact of living with ADHD’ theme, which highlight difficulties faced by people with ADHD in day-to-day life (e.g. self-neglect, self-care) and the relation to health. Similar themes around self-neglect and ADHD interfering with normal daily routines were identified in a Swedish study examining the lived experiences of those over 50 with ADHD [72, 100]. This turbulence in everyday life was also highlighted in a recent qualitative review of the life experiences of adults with ADHD, with findings suggesting that problems around self-care are common in ADHD and have a health impact [72].

This impact of living with ADHD may be mitigated by adequate healthcare services and knowledge, as has been previously reflected in qualitative lived experience work [72]. Some of this work may involve effective treatment of underlying ADHD [101], some may involve adapting existing services to be more accessible to people with ADHD, with the inaccessibility of healthcare being frequently referenced in studies looking at healthcare use in ADHD [46, 47, 72, 102]. However, some of the problems drawn out in qualitative analysis such as self-care/self-neglect may require an entirely novel approach, with further work needed to understand how to enable people with ADHD to take control of their health or develop self-efficacy, a concept which has previously been studied for example within behavioural interventions [30, 103–105].

The qualitative findings around sensation-seeking also highlight a key challenge of health risk prevention. Preventative care is largely dependent on people opting for a long-term benefit (e.g. living longer, better quality of life) over short-term reward (e.g. a ‘stim’, immediate emotional regulatory needs). In the case of ADHD and from what is understood about risk evaluation, current narratives may do little to successfully mitigate risk [89, 106, 107]. Further work examining how stakeholders may put more potential immediate positive benefits on not engaging in risky behaviour may be required. This work could perhaps relate to themes identified in the evolution of these health risks, for example sensation seeking and impulsivity. Providing balanced and well-communicated information to people with ADHD about potential wider health risks appears to be something people with ADHD would welcome [108], and might support better self-management of health and risks for this population.

There are several important clinical implications of this work. Firstly, this work provides evidence that the health risks in ADHD are important and pertinent to the daily lives of people with ADHD (especially for example in relation to substance misuse). Secondly, the findings of this work imply that the health problems related to ADHD are not conceptualised as separate to ADHD, but inter-related, necessitating a holistic approach which includes optimisation of ADHD management. This is particularly relevant within the context of our work, looking at the provisions and accessibility of care for people with ADHD.

### Strengths and limitations

The strengths of this study include a large sample with representation from various stakeholder groups across England, and the use of free text responses which allowed participants not only to ‘list’ risks but also to expand on their perspectives. The main limitation of the study is the ambiguity in the open question. Although partly intentional so participants did not feel restricted in what they could answer, it may have introduced elements of recall bias, with people recalling the most memorable and possibly extreme risks (e.g. substance misuse, homelessness) rather than those that may be more interpreted as more banal (e.g. smoking). Furthermore, the participants who gave more qualitatively rich answers that could be used for analysis may be a self-selecting subpopulation. Moreover, another important limitation would be the convenience sampling approach used, and the likely responder bias this introduces (respondents who have neutral attitudes towards ADHD would be less likely to respond than those with stronger views about ADHD in primary care). Due to the nature of the survey, we were unable to validate whether or not people had formal ADHD diagnoses, what the circumstances of that diagnosis were (e.g. late diagnosis) or whether they were self-identifying as having ADHD/awaiting diagnosis. This may also have impacted upon our results.

## Conclusion

This study set out a simple question; what are the most important health risks to stakeholders in ADHD? What this study provides is a complex answer. Quantitatively, the main health risks reported were substance use disorders, disordered eating, sleep disorders and weight/exercise management, with unprompted answers often concordant between at least two different stakeholders. Qualitatively, it seems that these health risks are perhaps perceived as manifestations of the difficulties of living with ADHD, which may be mitigated or exacerbated by other factors (i.e., support and awareness) and are highly heterogenous between participants. However, in UK national guidance there is a lack of reliable information for clinicians about what the physical health risks of ADHD may be or how to address them, and this may be rooted in the small evidence base in this field. More needs to be done to study both existing and novel interventions on the functional and health outcomes in ADHD, and to improve relevant healthcare information for healthcare professionals and people with ADHD on this topic, such that this significant health inequality can be addressed.

### Electronic supplementary material

Below is the link to the electronic supplementary material.

Supplementary Material 1

Supplementary Material 2

## Data Availability

The datasets used and/or analysed during the current study are available from the corresponding author on reasonable request. These data are not publicly available.

## Abbreviations

ADHD: Attention Deficit Hyperactivity Disorder
CMHT: Community Mental Health Team
GP: General Practice/Practitioners
MAP: Mapping ADHD in Primary Care
NICE: National Institute for Health and Care Excellence
NIHR: National Institute for Health Research
SPCR: School for Primary Care Research
UK: United Kingdom
US: United States
