# Supplementary material for: Patient, supporter and primary healthcare professional perspectives on health risks in over 16s with attention deficit hyperactivity disorder (ADHD) in England: a national survey study
Source: BMC Health Serv Res. 2024 Jun 19;24:751. doi: 10.1186/s12913-024-11188-5 (PMC11188530; doi:10.1186/s12913-024-11188-5)
Supplement: Supplementary file 1 — Supplementary Material 1 [file 12913_2024_11188_MOESM1_ESM.pdf]

UNIVERSITY OF EXETER | MEDICAL SCHOOL

MAP

Information

Primary Care Services for People with Attention Deficit Hyperactivity Disorder (ADHD)

Study title: Managing young people with ADHD in primary care (MAP) study

Chief Investigator: Dr Anna Price a.price@exeter.ac.uk

Website: https://sites.exeter.ac.uk/mapadhd/

We would like to invite you to take part in a national survey being undertaken at the University of Exeter. This research aims to find out about the healthcare services for people with ADHD that are available from GP practices. This research is funded by the National Institute for Health and Care Research (NIHR)

Please download and read: MAP study participant information sheet
This explains the aims of the study and what taking part would involve for you.

By taking part, you will help us gather valuable information which will be used to help improve healthcare for people with ADHD in the future. You will also have the chance to enter a prize draw for a £50 gift voucher, as a thank you!

Consent to continue

I have read the participant information sheet and wish to continue to the survey

- ○ Yes, take me to the survey
- ○ No, I do not wish to continue
- ○ Maybe, I would like more information

More information

What is the survey about?

This study aims to find out about primary care services for young people and adults with attention deficit hyperactivity disorder (ADHD) in England. Primary care services provide the first point of contact in the healthcare system, acting as the 'front door' of the NHS. Primary care staff, such as GPs, nurses, and pharmacists, play an important role in providing healthcare to people with ADHD. This includes referrals to secondary services, monitoring and prescribing ADHD medication, and providing psychological support. The aim of this survey is to map current service provision for adults with ADHD in England, so that we can improve and better co-ordinate primary care for young people aged 16-25 years with ADHD.

Why have I been approached to take part?

You have been approached to take part because we would like to hear from:
• people with ADHD (who are aged 16 or older), and their supporters (e.g., partners or family members), and
• primary healthcare professionals (such as GPs, nurses, and practice managers),
To take part you need to be aged 16 years or over and living or working in England.

What would taking part involve?

You will be asked to complete an online questionnaire that we anticipate will take 10-15 minutes to complete. The questions ask what you know about primary care (GP) services for ADHD in your area.

What are the possible benefits of taking part?

By taking part, you will help us gather valuable information which will be used to help improve healthcare for people with ADHD in the future. You will also have the chance to enter a prize draw for a £50 gift voucher, as a thank you!

Do I need to take part and what will happen if I don't want to carry on with the study?

No, you do not need to take part. It is entirely up to you to decide. This information sheet has been written to help you decide if you would like to take part or not. If you do decide to take part you are also free to change your mind at any time, without giving a reason and with no negative consequences.

*Once you have read and understood this information (and if you decide you wish to continue), please click the **NEXT** button*

Demographics 1 Stakeholder group

Please only complete this survey if you are **aged 16 years or over** and living or working in **England**, and also:

- A person with ADHD, *or*
- A supporter of a person (*aged 16 or over*) with ADHD*, or*
- A person working in NHS primary healthcare (*such as a GP, nurse, pharmacist, or practice manager*).

This survey is anonymous and confidential. It should take less than 15 mins to complete. We will not share any of your personal data.

Supporter - a family member, partner, or friend, who supports a person with ADHD

Are you...

Please choose whichever describes you best. You will have the chance to identify additional roles below.

- ○ A supporter of a young person or adult (aged 16 or over) with ADHD
- ○ A young person or adult with ADHD (aged 16 or over)
- ○ A person working in NHS primary care (e.g., GP, nurse, pharmacist, practice manager, administrator, or social prescriber)
- ○ None of the above, please take me to the end of the survey

Demographics 2 Role - YP

We would like to know about your 'main' role. Are you?

Please select whichever describes you best. You will have the chance to identify additional roles below.

- ○ A young person with ADHD (aged 16 or 17)
- ○ A young adult with ADHD (aged 18-25)
- ○ An adult with ADHD (aged 26 or older)

We would like to know about your 'main' role. Are you?

If you support more than one person with ADHD please check the box for the person you will answer questions about for this survey.

- ○ A supporter of a young person with ADHD (aged 16 or 17)
- ○ A supporter of a young adult with ADHD (aged 18-25)
- ○ A supporter of an adult with ADHD (aged 26 or older)

Are you also?

If any of the options below also describe you, please check all that apply

- ☐ A supporter of a child (aged under 15) with ADHD
- ☐ A supporter of a young person or adult (aged 16 or over) with ADHD
- ☐ A person working in NHS primary care (e.g., GP, nurse, pharmacist, practice manager, administrator, or social prescriber)
- ☐ None of these apply

Are you also?

If any of the options below also describe you, please check all that apply

- ☐ A supporter of a child (aged under 15) with ADHD
- ☐ A young person or adult with ADHD (aged 16 or over)
- ☐ A person working in NHS primary care (e.g., GP, nurse, pharmacist, practice manager, administrator, or social prescriber)
- ☐ None of these apply

Demographics 3 Location - YP

We would like to know about primary care provision from your GP practice (or the practice of the person you support).

Data will be treated confidentially and stored securely. Location data will only be used for the purposes of mapping areas where responses come from. For example, postcode data will be used to identify the relevant integrated care system, primary care network, or NHS region.

Your GP practice - *Your GP practice, or the practice of the person you support*

What is the postcode of your GP practice (if known), or of the home address that is local to this practice.

Enter with a space e.g., EX1 2LU [ ]

Core questions 2 Local provision - YP

Healthcare at your GP practice

We would like to know about healthcare and support provided for young people and adults with ADHD by staff at your GP practice. This question is about services provided by staff at your GP practice for someone with ADHD, but not healthcare received

directly from other NHS services, such as CAMHS or AMHS.

Young people and adults with ADHD - *Any young person with suspected or confirmed ADHD, aged from 16 years old and upwards*

Your GP practice - *Your GP practice, or the practice of the person you support*

Staff - *Staff located at your GP practice, such as your GP, a practice nurse, occupational therapist, care co-ordinator, or health and wellbeing coach*

CAMHS - Child and adolescent mental health services

AMHS - *Any adult mental health service including specialist ADHD teams*

Have **staff at your GP practice** provided you (or the person you support) with information to help understand and self-manage ADHD?

E.g., providing leaflets, or links to websites, video clips or podcasts

- Yes
- No
- Do not know
- Not applicable

Have **staff at your GP practice** ever provided you (or the person you support) with help and advice about moving between child and adult health services (transition)?

E.g., talking about what changes to expect in a routine appointment, or help with connecting to other services that you might need as an adult

- Yes
- No
- Do not know
- Not applicable

Have **staff at your GP practice** provided you (or the person you support) with mental health support?

E.g., through an appointment with a wellbeing practitioner at your local GP practice. This question is NOT asking about referrals made by your GP practice to another NHS mental health service

- Yes
- No
- Do not know
- Not applicable

Have **staff at your GP practice** signposted you (or the person you support) to other services such as charities or social care services?

E.g., providing contact details of ADHD support organisations, or helping you to contact social care services for support with living with ADHD. Please note, this is about signposting, but ***not*** *about referral to other NHS services, such as CAMHS or AMHS*

- Yes
- No
- Do not know
- Not applicable

Have **staff at your GP practice** provided you (or the person you support) with extra support with managing other health needs?

E.g., talking about possible risks of alcohol misuse or smoking, or helping with physical health screening like blood pressure checks

- Yes
- No
- Do not know
- Not applicable

Have **staff at your GP practice** linked you (or the person you support) with a social prescriber or 'community connector'?

E.g., a link worker who helps you to connect with a range of local (non-clinical) services like organised sports or social activities, to support health and wellbeing

- Yes
- No
- Do not know
- Not applicable

How would you rate the quality of healthcare provided by your GP practice for young people and adults with ADHD?

- Extremely good
- Moderately good
- Neither good nor bad

- Moderately bad
- Extremely bad
- Do not know

Core questions 3 Health risks - YP

Health risks

What do you consider to be the most important increased health risks linked with having ADHD?

This is not a knowledge test, we are interested in levels of awareness. Please list all those you consider to be important.

Have **staff at your GP practice** advised you (or the person you support) on managing any of the following?

We are interested in whether staff at your GP practice have provided targeted advice and support on any of these issues, which can be harder to manage when living with *ADHD*

- Sexual health & sexual health screening
- Stopping smoking & referral to smoking cessation services
- Physical activity & healthy eating
- Managing long term physical health conditions such as diabetes, epilepsy, or asthma
- Managing long term mental health conditions such as depression or anxiety
- Managing risky behaviours such as substance misuse (taking drugs)
- Other (please specify)
- Do not know
- This has not been provided

Core questions 4 Info resources - YP

We are interested in how different methods of sharing information can help with understanding and self-managing living with ADHD.

Information resources you use

Which of the following methods **do you (or does the person you support)** use to get information to help with understanding and self-managing living with ADHD? Also

which would you find useful?

Check all that apply

	Use currently	Would be useful
Websites	○	○
Printed material (book, leaflet, guide)	○	○
Audio clips or podcasts	○	○
Video clips	○	○
Digital app to help self-manage ADHD	○	○
Support groups (online or in person)	○	○
Conversation with a friend	○	○
Do not know	○	○

Please list resources you use or have found helpful when learning about managing the health needs of people living with ADHD.

For example, the ADHD Foundation resource hub (https://www.adhdfoundation.org.uk/resources/), or a YouTube channel. Please provide a link if possible.

Information from your GP practice

Which of the following types of resources have **staff at your GP practice** signposted or shared with you (or the person you support) to help with understanding and self-managing ADHD?

Check all that apply. This question is only asking about resources provided by your GP practice. It is not asking about information provided by other services such as CAMHS or AMHS.

	Have shared	Would be useful
Websites	○	○
Printed materials (book, leaflet, guide)	○	○
Audio clips or podcasts	○	○
Video clips	○	○
Digital app to help self-manage ADHD	○	○
Support groups (online or in person)	○	○
Do not know	○	○
None, they have not	○	○

Please list any resources your GP has shared with you (or the person you support) to provide information and support for people living with ADHD

For example the NHS website (https://www.nhs.uk/conditions/attention-deficit-hyperactivity-disorder-adhd/) or a local support group. Please provide a link if possible.

Future involvement

Thank you for taking the time to complete this survey. Would you like to be?

Check all that apply

- ☐ Entered into a prize draw for a £50 voucher
- ☐ Contacted about taking part in future research
- ☐ Kept informed about results of this research
- ☐ None of the above, please take me to the end of the survey

Please provide your contact details.

These will be stored securely and only used for the purposes you have indicated above

First name

Surname

Email address

Confirm email address

Core questions 1 AMHS - YP

Service links

We would like to know about how staff at your GP practice link you with other healthcare services, such as Adult Mental Health Services (AMHS) for patients with adult ADHD. Also about healthcare and support provided for young people and adults with ADHD at your GP practice.

Your GP practice - *Your GP practice, or the practice of the person you support*

AMHS - *Any adult mental health service including specialist ADHD teams*

Have **staff at your GP practice** ever referred you (or the person you support) to an Adult Mental Health Service (AMHS) or adult ADHD service for healthcare provision for

ADHD?

To provide diagnosis and/or management of adult ADHD, as needed and in line with NICE guidelines [NG87]

- Yes
- No
- Do not know

Roughly how long is the waiting list for patients referred to this AMHS service?

- 0 months, up to 6 months
- 6 months, up to 12 months
- 12 months, up to 24 months
- 2 years or more
- Do not know

How would you rate the quality of healthcare for adult patients with ADHD **provided by this AMHS**?

This question is asking about the quality of healthcare from the adult mental health service for adult ADHD, not from your GP practice.

- Extremely good
- Moderately good
- Neither good nor bad
- Moderately bad
- Extremely bad
- Do not know

Have **staff at your GP practice** provided you (or the person you support) with prescriptions for adult ADHD medications following an NHS diagnosis for ADHD?

E.g., repeat prescriptions of adult ADHD medication as a part of a shared care agreement with an adult mental health service

- Yes
- No
- Do not know
- Not applicable

Have **staff at your GP practice** provided you (or the person you support) with prescriptions for adult ADHD medications following a PRIVATE (non-NHS) diagnosis?

E.g., provided repeat prescriptions of adult ADHD medication

- Yes
- No
- Do not know
- Not applicable

Have **staff at your GP practice** provided any of the following, so that they can start (or continue) prescribing adult ADHD medication?

Check all that apply

- Monitoring of blood pressure and pulse
- Monitoring of weight
- Undertaking blood tests as required, e.g., liver function test
- Do not know
- Not applicable

Demographics 2 Role - P

We would like to know about your 'main' primary care related role. Please select whichever describes you best, including if you are a trainee.

You will have the chance to identify additional roles below

- General Practitioner
- Nurse
- Pharmacist
- Manager or administrator
- Other role in primary care (e.g., nursing associate, or mental health practitioner)
- ________ Other (please specify)

Which 'other role in primary care' do you hold?

- Care co-ordinator
- Health and wellbeing coach
- Pharmacy technician
- Occupational therapist

- ○ Physician associate
- ○ Nursing associate
- ○ Social prescribing link worker
- ○ Mental health practitioner or counsellor
- ○ [ ] Other (please specify)

Are you also?

If any of the options below also describe you, please check all that apply.

- ☐ A young person or adult with ADHD (aged 16 or over)
- ☐ A supporter of a young person or adult (aged 16 or over) with ADHD
- ☐ A supporter of a child (aged under 15) with ADHD
- ☐ [ ] Other (please specify)
- ☐ None of the above

Demographics 3 Location - P

We would like to know about primary care provision local to where you work.

Data will be treated confidentially and stored securely. Location data will only be used for the purposes of mapping areas where responses come from. For example, postcode data will be used to identify the relevant integrated care system, primary care network, or NHS region.

What is the postcode of the primary care (GP) practice you will be answering questions about.

If you work in more than one practice or across a network, please choose the location you are most familiar with

Enter with a space e.g., EX1 2LU [ ]

Core questions 1 AMHS - P

Support and advice for primary care

We would like to know about support and advice available to staff at your GP practice from Adult Mental Health Services (AMHS) for patients with adult ADHD.

Your GP practice - *The primary care practice or network in which you work. Or if you work with several practices, the one you work most closely with.*

AMHS - *Any adult mental health service including specialist ADHD teams*

Is there an NHS funded AMHS, with staff with expertise in ADHD, that your practice can refer patients to?

To provide the patient with diagnosis and/or management of adult ADHD, as needed and in line with NICE guidelines [NG87]

- Yes
- No
- Do not know
- Not applicable

Roughly how long is the waiting list for patients referred to this service?

- 0 months, up to 6 months
- 6 months, up to 12 months
- 12 months, up to 24 months
- 2 years or more
- Do not know

How would you rate the quality of healthcare for adult patients with ADHD provided by this AMHS?

- Extremely good
- Moderately good
- Neither good nor bad
- Moderately bad
- Extremely bad
- Do not know

Do staff at your practice have access to advice (or an 'advice and guidance' service) from AMHS healthcare professionals with specialist knowledge of adult ADHD?

For example, someone available on the phone to provide advice on the management of a patient with adult ADHD within primary care

- Yes
- No
- Do not know
- Not applicable

Does your practice have shared care protocols/agreements in place with an AMHS to enable prescribing of adult ADHD medications through primary care?

ADHD medications - NICE guideline [NG87] recommended medications for adult ADHD.

- Yes
- No
- Do not know
- Not applicable

How well is your practice supported by AMHS, to manage the healthcare needs of adult patients with ADHD?

- Extremely supported
- Moderately supported
- Neither supported or unsupported
- Moderately unsupported
- Extremely unsupported
- Do not know
- Not applicable

Does your practice prescribe adult ADHD medications for patients with an NHS diagnosis of ADHD?

NHS diagnosis - *With an NHS diagnosis for adult ADHD*

- No
- Yes
- Do not know
- Not applicable

How does your practice manage prescribing ADHD medications for adult patients with an NHS diagnosis of ADHD?

- Without a shared care agreement
- With a shared care agreement with NHS AMHS
- ______ Other (please specify)
- Do not know

Does your practice prescribe adult ADHD medications for patients with only a private diagnosis of ADHD?

Private diagnosis - *With a private diagnosis for adult ADHD, without an NHS diagnosis, and not currently registered with an NHS adult service for their ADHD*

- ○ No
- ○ Yes
- ○ Do not know
- ○ Not applicable

How does your practice manage prescribing adult ADHD medications for patients with only a private diagnosis of ADHD?

- ○ Without a shared care agreement
- ○ With a shared care agreement with a Private Provider
- ○ [ ] Other (please specify)
- ○ Do not know

What do you or your practice provide (undertake to do) as part of a shared care agreement, to enable safe prescribing of adult ADHD medication?

Check all that apply

- ☐ Monitoring of blood pressure and pulse
- ☐ Monitoring of weight
- ☐ Undertaking blood tests as required, e.g., liver function test
- ☐ Not applicable

Core questions 2 Local provision - P

Provision in Primary Care

We would like to know about the support available from your GP practice for young people and adults with ADHD. This includes support provided through the primary care network, but not support from other services, such as secondary care, or voluntary sector organisations.

Young people and adults with ADHD - *Any young person with suspected or confirmed ADHD, aged from 16 years old and upwards*

Which of the following services do staff at your GP practice (or within the local primary care network) provide to young people and adults with ADHD?

Check all that apply

- ☐ Referral to secondary NHS services
- ☐ Prescriptions for adult ADHD medication (patient with NHS diagnosis)
- ☐ Prescriptions for adult ADHD medication (patient with PRIVATE diagnosis)
- ☐ Providing (or signposting to) information to help understand & self-manage ADHD
- ☐ Support for transition from child to adult services, e.g. advising on expected changes, or linking to a transition worker
- ☐ Mental health support directly from primary care, for example via a wellbeing coach
- ☐ Signposting to other services, such as charities, or social care
- ☐ Extra and targeted support with managing other health needs
- ☐ Social prescribing, e.g. link workers connecting patients with local (non-clinical) services like sports or social activities, to support health & wellbeing
- ☐ Not applicable
- ☐ None, not provided

How would you rate the quality of healthcare provided by staff at your primary care practice for young people and adults with ADHD?

- ○ Extremely good
- ○ Moderately good
- ○ Neither good nor bad
- ○ Moderately bad
- ○ Extremely bad
- ○ Do not know
- ○ Not applicable

Core questions 3 Health risks - P

Health risks

What do you consider to be the most important increased health risks associated with having ADHD?

This is not a knowledge test, we are interested in levels of awareness. Please list all those you consider to be important

Do you or staff at your practice give targeted advice (or support) on any of the following as part of your approach to supporting patients with ADHD?

- ☐ Sexual health & sexual health screening
- ☐ Smoking cessation & referral to smoking cessation services
- ☐ Physical activity & healthy eating
- ☐ Managing long-term physical health conditions e.g., diabetes
- ☐ Managing long-term mental health conditions e.g., depression
- ☐ Managing risky behaviours such as substance misuse
- ☐ ____________ Other (please specify)
- ☐ Do not know
- ☐ Not provided

Core questions 4 Info resources - P

We are interested in different methods staff at your GP practice use to gain information to help **both staff and patients** with understanding and managing ADHD.

Information for staff

Which of the following types of resources do **staff at your practice** use, and which would they find useful, to help them manage the healthcare needs of patients with ADHD?

Check all that apply

	Use currently	Would be useful
Websites	☐	☐
Printed material (book, leaflet, guide)	☐	☐
Audio clips or podcasts	☐	☐
Video clips	☐	☐
Online training	☐	☐
Digital app to guide decision making	☐	☐
Conversation with a colleague	☐	☐
Do not know	☐	☐

Please list resources you use or have found helpful when managing the health needs of people living with ADHD.

For example NICE guideline [NG87] on diagnosis and management of ADHD (https://www.nice.org.uk/guidance/ng87), or a locally available resource such as a shared care agreement. Please provide a link if possible.

Information for patients

Which of the following resources do staff at your practice **signpost or share with patients** to help them to gain information to understand and self-manage their ADHD? Also, which of these would they find useful if available?

Check all that apply

	Use currently	Would be useful
Websites	☐	☐
Printed materials (book, leaflet, guide)	☐	☐
Audio clips or podcasts	☐	☐
Video clips	☐	☐
Digital apps to help self-manage ADHD	☐	☐
Online training	☐	☐
Support groups (online or in person)	☐	☐
Do not know	☐	☐

Please list resources you use or have found helpful to signpost or share with patients with ADHD.

For example, the ADHD Foundation resource hub (https://www.adhdfoundation.org.uk/resources/) or a local support group.

FUNDED BY
NIHR | National Institute for Health Research

Powered by Qualtrics

UNIVERSITY OF EXETER | MEDICAL SCHOOL

MAP

Information - C

Primary Care Services for People with Attention Deficit Hyperactivity Disorder (ADHD)

Study title: Managing young people with ADHD in primary care (MAP) study

Chief Investigator: Dr Anna Price a.price@exeter.ac.uk

Website: https://sites.exeter.ac.uk/mapadhd/

This national survey is being undertaken at the University of Exeter. This research aims to find out about the healthcare services for people with ADHD that are available through primary care. The research is funded by the National Institute for Health and Care Research (NIHR) and has NHS Health Research Authority (HRA) ethical approval from the Bradford Leeds Research Ethics Committee (22/YH/0132).

By completing this freedom of information request (FOI), you will help to gather valuable information to help improve healthcare for people with ADHD. Findings may inform the development of innovative solutions to provide joined up care and support for people with ADHD through integrated care systems (ICSs), and help to tackle health inequalities.

Demographics 3 Location - C

We would like to know about commissioning arrangements for young people and adults with ADHD provided by integrated care boards (ICBs) across England, with a focus on primary care.

Which NHS region is your Integrated Care Board (ICB) located within?

- ○ East of England
- ○ London

- Midlands
- North East and Yorkshire
- North West
- South East
- South West

Which ${q://QID229/ChoiceGroup/SelectedChoices} ICB do you represent?

Note, the following questions may refer either to the ICB and/or the integrated care system (ICS) area that it represents

- Bedfordshire, Luton and Milton Keynes
- Cambridgeshire and Peterborough
- Hertfordshire and West Essex
- Mid and South Essex
- Norfolk and Waveney
- Suffolk and North East Essex

Which ${q://QID229/ChoiceGroup/SelectedChoices} ICB do you represent?

Note, the following questions may refer either to the ICB and/or the integrated care system (ICS) area that it represents

- North Central London
- North East London
- North West London
- South East London
- South West London

Which ${q://QID229/ChoiceGroup/SelectedChoices} ICB do you represent?

Note, the following questions may refer either to the ICB and/or the integrated care system (ICS) area that it represents

- Birmingham and Solihull
- Black Country
- Coventry and Warwickshire
- Derby and Derbyshire
- Herefordshire and Worcestershire
- Leicester, Leicestershire and Rutland

- Lincolnshire
- Northamptonshire
- Nottingham and Nottinghamshire
- Shropshire, Telford and Wrekin
- Staffordshire and Stoke-on-Trent

Which ${q://QID229/ChoiceGroup/SelectedChoices} ICB do you represent?

Note, the following questions may refer either to the ICB and/or the integrated care system (ICS) area that it represents

- Humber and North Yorkshire
- North East and North Cumbria
- South Yorkshire
- West Yorkshire

Which ${q://QID229/ChoiceGroup/SelectedChoices} ICB do you represent?

Note, the following questions may refer either to the ICB and/or the integrated care system (ICS) area that it represents

- Cheshire and Merseyside
- Greater Manchester
- Lancashire and South Cumbria

Which ${q://QID229/ChoiceGroup/SelectedChoices} ICB do you represent?

Note, the following questions may refer either to the ICB and/or the integrated care system (ICS) area that it represents

- Buckinghamshire, Oxfordshire and Berkshire West
- Frimley
- Hampshire and Isle of Wight
- Kent and Medway
- Surrey Heartlands
- Sussex

Which ${q://QID229/ChoiceGroup/SelectedChoices} ICB do you represent?

Note, the following questions may refer either to the ICB and/or the integrated care system (ICS) area that it represents

- Bath and North East Somerset, Swindon and Wiltshire
- Bristol, North Somerset and South Gloucestershire
- Cornwall and The Isles of Scilly
- Devon
- Dorset
- Gloucestershire
- Somerset

Please provide your details

Job role	
Name	
Email address	
Phone number	

Please provide details of the person (if not you) within your ICB that is responsible for commissioning services to support the delivery of primary healthcare for young people and adults with ADHD.

For example we are interested in:

- *Mental health and social prescribing*
- *Shared care arrangements*
- *Other locally enhanced services that may affect care provided for young people and adults with ADHD*

Job role	
Name	
Email address	
Phone number	
Comments	

Core questions 1 AMHS - C

Adult mental health services for ADHD

We would like to know about the healthcare available from adult mental health services (AMHS) for young people and adults with ADHD that are located in your ICS

AMHS - Any adult mental health service including specialist ADHD teams

Young people and adults with ADHD - Any young person with suspected or confirmed ADHD, aged from 16 years old

and upwards

Is there an NHS funded AMHS that is available to provide healthcare (diagnosis and/or management) of adult ADHD for patients within your ICS?

- Yes
- No
- Do not know
- Not applicable

Roughly how long is the waiting list for patients referred to this service?

- 0 months, up to 6 months
- 6 months, up to 12 months
- 12 months, up to 24 months
- 2 years or more
- Do not know

In your ICB, can primary care providers refer patients using the 'right to choose' legislation to other adult healthcare services for ADHD (outside of your ICS footprint)?

For example, commissioned private adult ADHD services that are available online.

- Yes
- No
- Do not know
- Not applicable

If you would like to add anything on the 'right to choose' legislation, please comment below

Do primary care providers in your ICS have shared care protocols/agreements in place with an AMHS to enable prescribing of adult ADHD medications through primary care?

ADHD medications - NICE guideline [NG87] recommended medications for adult ADHD.

- Yes

- ○ No
- ○ Do not know
- ○ Not applicable

If you would like to add anything on shared care provision for young people with ADHD, please comment below.

[ ]

Core questions 2 Local provision - C

Primary Care services for ADHD

We would like to know about the support provided by primary care in your ICS for young people and adults with ADHD.

This includes support from primary care providers or networks (PCNs), but not support from other services, such as secondary care, or voluntary sector organisations

Are any of the following additional roles being funded within your ICS (either via PCNs or directly)

Please check all that apply, including for roles that are in development.

- ☐ Care co-ordinator
- ☐ Health and wellbeing coach
- ☐ Pharmacy technician
- ☐ Occupational therapist
- ☐ Physician associate
- ☐ Nursing associate
- ☐ Social prescribing link worker
- ☐ Mental health practitioner or counsellor
- ☐ [ ] Other (please specify)

Please indicate whether these these roles have been filled (in place), are still in development (in progress) or if this varies by provider/PCN (mixed).

	In place	In progress	Mixed	Comments
» Care co-ordinator	○	○	○	[ ]

	In place	In progress	Mixed	Comments
» Health and wellbeing coach	○	○	○	
» Pharmacy technician	○	○	○	
» Occupational therapist	○	○	○	
» Physician associate	○	○	○	
» Nursing associate	○	○	○	
» Social prescribing link worker	○	○	○	
» Mental health practitioner or counsellor	○	○	○	
» Other (please specify)	○	○	○	

Does your ICB have any enhanced services agreed with primary care providers for prescribing and monitoring of ADHD medication?

This question is asking about agreements to provide additional care (in place or in development), but not about shared care.

- ☐ Yes
- ☐ No
- ☐ Do not know
- ☐ Not applicable

Does your ICB have any other enhanced services agreed with primary care providers for extended support or services for people with long term mental health conditions (such as ADHD)?

This question is asking about agreements to provide additional care (in place or in development), but not about shared care.

- ☐ Yes
- ☐ No
- ☐ Do not know
- ☐ Not applicable

Please list the enhanced services agreed with primary care providers in your ICS (that are relevant for people with ADHD).

If you would like to add anything about enhanced services, please comment below.

Has your ICB identified any localities within your ICS where there are inequalities in access to healthcare services for young people and adults with ADHD?

- ○ Yes
- ○ No
- ○ Do not know
- ○ Not applicable

If you would like to add anything about inequalities, please comment below.

Please list any barriers your ICB faces when commissioning effective services for young people (aged 16-25) with ADHD

If you would like to add anything to your answers, please comment below

Future involvement

Thank you for taking the time to complete this survey. Would you like to be?

Check all that apply

- ☐ Contacted about taking part in future research
- ☐ Kept informed about results of this research
- ☐ None of the above, please take me to the end of the survey

Please provide your contact details.

These will be stored securely and only used for the purposes you have indicated above

First name	
Surname	
Email address	
Confirm email address	

FUNDED BY
NIHR | National Institute for Health Research

Powered by Qualtrics
