# Supplementary material for: Patient, supporter and primary healthcare professional perspectives on health risks in over 16s with attention deficit hyperactivity disorder (ADHD) in England: a national survey study
Source: BMC Health Serv Res. 2024 Jun 19;24:751. doi: 10.1186/s12913-024-11188-5 (PMC11188530; doi:10.1186/s12913-024-11188-5)
Supplement: Supplementary file 2 — Supplementary Material 2 [file 12913_2024_11188_MOESM2_ESM.docx]

**Supplementary Material 1:**

The below questions, pertaining to health risks, have been extracted from the larger survey (Supplementary Material 2).

**Health risk questions to those with lived experience as presented in the survey.**

Q6.1 Health risks 
What do you consider to be the most important increased health risks linked with having ADHD? 
*This is not a knowledge test, we are interested in levels of awareness. Please list all those you consider to be important.*

Q6.2 Have **staff at your GP practice** advised you (or the person you support) on managing any of the following?  
We are interested in whether staff at your GP practice have provided targeted advice and support on any of these issues, which can be harder to manage when living with ADHD

- Sexual health & sexual health screening
- Stopping smoking & referral to smoking cessation services
- Physical activity & healthy eating
- Managing long term physical health conditions such as diabetes, epilepsy, or asthma
- Managing long term mental health conditions such as depression or anxiety
- Managing risky behaviours such as substance misuse (taking drugs)
- Other (please specify)
- Do not know
- This has not been provided

**Health risk questions to healthcare professionals as presented in the survey.**

Q14.1 Health risks 
What do you consider to be the most important increased health risks associated with having ADHD? 
*This is not a knowledge test, we are interested in levels of awareness. Please list all those you consider to be important*

Q14.2 Do you or staff at your practice give targeted advice (or support) on any of the following as part of your approach to supporting patients with ADHD?   

- Sexual health & sexual health screening
- Smoking cessation & referral to smoking cessation services
- Physical activity & healthy eating
- Managing long-term physical health conditions e.g., diabetes
- Managing long-term mental health conditions e.g., depression
- Managing risky behaviours such as substance misuse
- Other (please specify)
- Do not know
- Not provided
